# Comparison of Different Restoration Techniques for Endodontically Treated Teeth

**DOI:** 10.1155/2022/6643825

**Published:** 2022-02-11

**Authors:** Jusuf Lukarcanin, İsmail Serhat Sadıkoğlu, Bilal Yaşa, Lezize Şebnem Türkün, Murat Türkün

**Affiliations:** ^1^Medicana International Izmir Hospıtal, Department of Dentistry, Izmir, Turkey; ^2^European University of Lefke, Faculty of Dentistry, Restorative Dentistry Department, Lefke, Mersin10, Turkey; ^3^Izmir Katip Celebi University, School of Dentistry, Department of Restorative Dentistry, Izmir, Turkey; ^4^Ege University, School of Dentistry, Department of Restorative Dentistry, Izmir, Turkey

## Abstract

The aim of the present study is to evaluate the physical properties of endodontically treated teeth restored with five different restorative techniques and materials. Hundred and forty extracted human molar teeth were used. In addition to five restoration groups, specimens with no restorations were used as the negative control, and intact molar teeth were used as the positive control. For flexural strength tests, material specimens were made from 5 different materials using a mould according to ISO 4049 standards. One-way ANOVA revealed that the fracture resistance was significantly affected by the restoration type. SFRC group showed the best fracture resistance values, while lowest values were seen in the GWF group. The test results of flexural strength showed values between 140 and 184 MPa and modulus of elasticity between 6.33 and 18.89 GPa (*p* < 0.05). Under the limits of this study, results showed that SFRC can be used to increase the fracture resistance of ETT.

## 1. Introduction

One of the primary reasons for the extraction of endodontically treated teeth (ETT) is the formation of nonrestorable fractures in the coronal parts of the teeth [1]. This is attributed to the difference in the biomechanical properties between ETT and vital teeth [2, 3]. In this respect, coronal restoration of ETT has great importance in increasing the survival rate of these teeth [4]. Posterior resin composites have become preferred materials in coronal restorations because of their sufficient features such as applicable in a single session and having satisfactory aesthetic and mechanical properties. Fracture resistance of the teeth increases with the application of composite resins. However, in the presence of teeth with excessive substance loss, mechanical properties of coronal restorations should be strengthened [5].

Generally, composite restorations are applied as thin layers and polymerised separately due to the limited depth of cure of most conventional resin composites [6]. Also, another application reason of the incremental technique is to reduce the polymerisation shrinkage of composites [7, 8]. Bulk-fill composites, which can be applied and polymerised in a single layer of 4-5 mm thickness, are separated from conventional composites with their increased depth of cure properties [9]. With these features, bulk-fill composites reduce the negative aspects of polymerisation such as polymerisation shrinkage and shrinkage stresses more successfully than conventional composites [10, 11]. Therefore, bulk-fill composites are recommended to be used in deep and narrow cavities deeper than 4 mm, such as postendodontic restorations, instead of conventional composites [9].

In recent years, fibre-reinforced composites have been used in the restoration of teeth, especially in high occlusal stress areas [12]. Restoration strengthening with fibres can be done by various methods. One of them is using shortened fibre-reinforced composite (SFRC), which is developed for strengthening the coronal restoration under conventional composites. It has been reported that shortened fibres in the SFRC prevent the crack or fracture from moving along the tooth and act as a load barrier against high occlusal forces [13]. It has been demonstrated in several studies that using SFRC has increased restoration resistance compared to restorations done with conventional composites [13–16]. Another method of strengthening restorations with utilizing fibres is the use of woven fibres. Research studies showed that it is benefited to support the restorations with woven fibres, especially in ETT with excessive substance loss [17, 18]. Kemaloglu et al. [13] claimed that the use of fibre-reinforced restorations in ETT was more successful than conventional composites in terms of fracture strength.

The direct applications of resin composite materials have significant advantages for the patient and the physician; however, they also have disadvantages such as their polymerisation shrinkage. Furthermore, their mechanical properties may be insufficient, especially in high-stress areas such as posterior teeth exposed to chewing forces [19]. It has been reported that the problems such as microleakage, postoperative sensitivity, secondary caries, and the difficulties in providing ideal contact and contour in teeth with extensive substance loss can be reduced by applying indirect restorations that can be polymerised, finished, and polished outside the mouth, and successful results can be obtained [20].

The clinical success of a restorative application is directly related to the physical and mechanical properties of the material used. Mechanical tests are used to determine these mechanical properties. ISO (International Organisation for Standardisation) standards have been accepted as the standard test technique to determine the physical and mechanical properties of a material [21, 22]. Therefore, the aim of this in vitro study is to evaluate 5 different restoration methods for ETT and materials used in these methods comparatively. The null hypothesis of this study is that there will be no significant difference between the 5 techniques examined.

## 2. Materials and Methods

### 2.1. Sample Preparation for Fracture Strength Analysis

**Figure 1:**
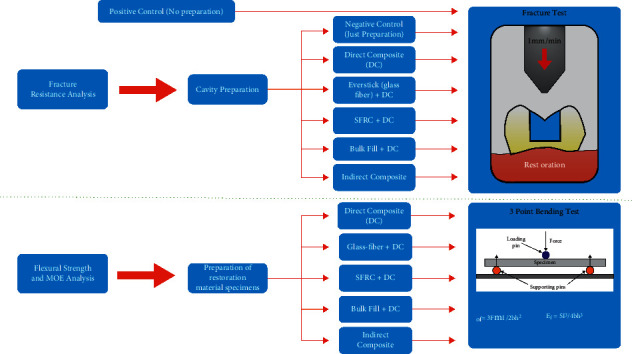
Schematic work flow of the research.

The ethics committee approval report was taken from the Ege University Faculty of Medicine Clinical Research Ethics Committee (no. 14–10/15) before the study. The schematic work flow of the study is presented in Figure 1. One hundred and forty caries-free, third molars were divided into 7 groups (*n* = 20), consisting of 5 restoration groups using different restoration techniques, a negative control (including teeth that were endodontically treated but were not restored), and a positive control (no treatment was applied). MOD cavities were prepared with the thickness of the buccal and lingual walls at the level of the equator line which was 2.5 mm ± 0.2 mm in order to ensure standardisation in all teeth except the positive control group. The gingival finish line on the proximal surface of the cavities was prepared 1 mm above the cementoenamel junction. Following endodontic access cavity preparation, all teeth were instrumented using ProTaper rotary files (Dentsply Maillefer, Ballaigues, Switzerland). During preparation, the root canals were irrigated with 2 ml of 2.5% sodium hypochlorite between each file. After completing the instrumentation, 5 ml of 5% EDTA, 5 ml of 2.5% NaOCl, and distilled water were used for the final irrigation, and all teeth were obturated with gutta-percha and AH Plus sealer (Dentsply DeTrey, Konstanz, Germany) using the single-cone technique. While proceeding to the restoration phase, following the application of 35% orthophosphoric acid (K-Etchant, Kuraray Noritake, Okayama, Japan) to the teeth in all restoration groups, the single-bottle adhesive system (G-aenial Bond, GC Corp., Tokyo, Japan) was applied and polymerised with the LED light-curing device (Elipar FreeLight 2, 3M ESPE, St. Paul, MN, USA) according to the manufacturer instructions. The cavities were then restored as follows:

- Group 1 (DC): initially, the missing proximal wall was restored with 1 mm-thick nanohybrid resin composite (G-aenial Posterior, GC Corp., Tokyo, Japan) using the Adapt SuperCap matrix system (Kerr, KerrHawe, Bioggio, Switzerland). After the proximal wall was formed, the matrix was removed, and the rest of the cavity was restored using the same composite, with the incremental technique as 2 mm-thick layers.
- Group 2 (GWF): after creating the proximal wall as described in group 1, a thin layer of flowable resin composite (FRC) (G-aenial Universal Flo, GC Corp., Tokyo, Japan) was applied into the cavity. Afterwards, a piece of glass woven fibre (GWF) (Everstick NET, GC Corp., Tokyo, Japan) with 8 mm length and 3 mm width was cut and placed in this composite, and the first fibre piece was placed in a buccolingual direction to be in close contact with the buccal and lingual walls and was cured for 20 s. After applying a thin layer of FRC again, the second piece of GWF was placed on the uncured FRC perpendicular to the first piece, covering the mesial and distal walls, and was cured for 20 s. The rest of the cavity was restored with nanohybrid composite resin (G-aenial Posterior, GC Corp., Tokyo, Japan) again with the incremental technique.
- Group 3 (SFRC): after creating the proximal wall as described in group 1, shortened fibre-reinforced resin composite (SFRC) (EverX Posterior, GC Corp., Tokyo, Japan) was applied to the cavity with 4 mm thickness. The remaining 2 mm deep part of the cavity was restored using nanohybrid composite resin (G-aenial Posterior, GC Corp., Tokyo, Japan).
- Group 4 (FBFC): after the etching and adhesive procedures, the ring matrix system Adapt SuperCap (Kerr, KerrHawe, Bioggio, Switzerland) was placed. Unlike other groups, 4 mm-thick SDR bulk-fill (Dentsply, Konstanz, Germany) was applied to the entire cavity at one time, without forming a proximal wall, and was polymerised with light. The remaining occlusal 2 mm part of the cavity was restored with nanohybrid composite resin G-aenial Posterior.
- Group 5 (IC): first of all, cavity impression was taken with Vinyl Polyether Silicone (VPES^TM^) GC EXA'lence (GC Corp., Tokyo, Japan) impression material in this group. After preparing working models from hard plaster, indirect restorations were formed using indirect composite resin (GC GRADIA, GC Corp., Tokyo, Japan). Formed restorations were polymerised in an indirect composite curing oven (GC Labolight LV-III, GC Corp., Tokyo, Japan) for 10 minutes. Finally, the restorations were cemented to the cavities using an adhesive cement (G-Cem, GC Corp., Tokyo, Japan).

### 2.2. Fracture Resistance Test

After the endodontic and restorative procedures were completed, teeth in all groups, including the control groups, were embedded into autopolymerising polymethyl methacrylate, up to 1 mm apical of the cementoenamel junction, using cylindrical moulds. All samples were kept in distilled water at room temperature for one week until the fracture test. Finally, the specimens were placed in a universal testing machine (Autograph AG-5 kNG, Shimadzu, Tokyo, Japan) for the axial compression test. The compression load was applied at a speed of 1 mm per min on the occlusal surface of the restoration and in contact with the cusps being parallel to the long axis of the tooth until a fracture occurred. The load resulting in tooth fracture was recorded. The fractured specimens were then removed from acrylic resin and assessed for fracture patterns. Repairable fractures above the level of the simulated bone were defined as “favorable failures,” whereas unrepairable fractures below this line were defined as “unfavourable failures.”

### 2.3. Sample Preparation for Flexure Strength and Modulus of Elasticity Tests

In addition to the mechanical properties of the restorations, in order to evaluate the flexural strength and elasticity module of the restoration materials, a total of 75 samples with 2 × 2 × 25 mm dimensions were prepared in accordance with ISO 4049 standards (*n* = 15). The samples in the flexural strength and elasticity module evaluation groups were prepared as follows:

- Group 1: the Mylar strip tape was placed on the bottom of the 2 × 2 × 25 mm-sized mould placed on a slide. A nanohybrid composite (G-aenial Posterior, GC Corp., Tokyo, Japan) was placed in the mould and slightly pressed by placing another strip, and slide on it again. After removing the excess material, polymerisation was achieved by applying 20 seconds of light from three points of the slide each, two edges, and the middle part of the mould. Then, the slide was removed, and 20 seconds of light was applied to the same areas again. Therefore, the total polymerisation time was increased to 120 seconds.
- Group 2: flowable composite (GC Universal Flo, GC Corp, Tokyo, Japan) was applied to the mould prepared as described in group 1, but it was not polymerised since the GWF was cut to size 24 × 2 mm and placed in the bottom of the mould. Then, nanohybrid resin was applied into the mould, and the application was completed as described in group 1.
- Group 3: application steps described in group 1 were applied in the same way, but SFRC (EverX Posterior, GC Corp., Tokyo, Japan) was used instead of the conventional nanohybrid composite.
- Group 4: application steps described in group 1 were applied in the same way, but a bulk-fill resin composite (SDR, Dentsply, Konstanz, Germany) was used instead of the conventional nanohybrid composite.
- Group 5: indirect composite resin samples were placed as described in the previous groups, and their initial polymerisation was completed. Subsequently, samples were placed in an indirect composite curing oven for 10 minutes in order to ensure final polymerisation.

### 2.4. Three-Point Bending Test and Calculation of Flexure Strength and Modulus of Elasticity Values

All samples were placed in distilled water at 37°C for 24 hours before subjected to a three-point bending test. After the testing apparatus has been installed, the universal testing machine was running at a speed of 0.5 mm/minute. The maximum force values causing the fracture were determined. The following formulas were used to calculate the numerical values of flexural strength and elasticity module.

(1) $$\begin{array}{c} o_{f}=\frac{3FmI}{2bh^{2}},\\ E_{f}=\frac{SI^{3}}{4bh^{3}}. \end{array}$$

### 2.5. Statistical Analysis

Statistical analysis of the study was done with the SPSS package program (IBM Statistics, Illinois, USA), and definitive statistics were obtained for all data. The Kolmogorov–Smirnov and Levene's tests examined the normality of the distribution and the homogeneity of the variance. Group comparisons were made with one-way ANOVA, and binary comparisons between groups were evaluated by the post hoc Tukey HSD test. For all tests, *p*=0.05 value was considered significant.

## 3. Results

### 3.1. Fracture Strength and Failure Mode Results

The average fracture strength values and standard deviations of the groups are shown in Table 1. Among the restoration groups, the highest fracture resistance was observed in the SFRC group. While the SFRC group showed significantly higher results than the GWF group and the indirect composite group, no significant difference was observed between bulk-fill and direct composite restoration groups. The lowest fracture resistance was observed in the GWF group. However, no significant difference was found among GWF, IC, and DC groups. When the fracture resistance data of this study were examined, an 85% reduction was found in the fracture resistance of the negative control group, compared to the positive control group. This decrease was found to be at the level of 38% in the GWF group, 30% in the indirect composite group, 28% in the direct composite group, 25% in the bulk-fill group, and 18% in the SFRC group.

When failure modes were analysed, a total of 36 samples from 100 samples tested in restoration groups were classified as favorable, and 64 samples were classified as unfavorable. The distribution of favorable and unfavorable fracture percentages of groups is shown in Table 2.

### 3.2. Flexural Strength and Modulus of Elasticity Results

The flexural strength and elasticity modules of the restoration groups are presented in Tables 3 and 4, respectively. When the flexural strength data were examined, the highest flexural strength was observed in the SFRC group. The flexural strength values observed in the SFRC group were found to be significantly higher than those of the direct and indirect composite groups (*p* < 0.05), despite no significant difference was found between the SFRC and FBFC groups (*p*=0.737). While the lowest flexural strength values were observed in the GWF group, no statistically significant difference was found between GWF and direct (*p*=0.775) and indirect (*p*=0.733) composite groups.

In terms of the modulus of elasticity, the SFRC group was found to be significantly higher compared to other groups (*p* < 0.05). The lowest modulus of elasticity was observed in the FBFC group, but the difference between this group and the indirect composite group was not significant (*p*=0.61).

## 4. Discussion

Direct restorations are highly preferred because they can be applied in a single appointment and relatively economical. However, direct restorations also show a great variety in themselves due to new materials that are constantly developing and coming to the market. Indirect restorations are prepared outside the oral cavity and can be produced with various materials such as composites and porcelain. It is difficult for clinicians to make the right choice in the presence of so many materials and restoration preferences. Therefore, fracture resistance evaluation of five popular restoration techniques and flexure resistance and modulus of elasticity evaluation of materials which are used in these techniques have been done and compared in this study.

Belli et al. [23] stated that MOD cavity preparation caused a decrease in fracture strength. Reeh et al. [24] reported that the fracture resistance of ETT with an occlusal cavity was decreased only by 5%, and when the MOD cavity was prepared, the fracture strength decreased by 69%. Similar to the mentioned studies, in this study, it was determined that there was an 85% reduction in the fracture strength of the teeth with the MOD cavity compared to the teeth that did not prepare.

Özşevik et al. [25] showed that the fracture resistance of the teeth restored with fibre-reinforced composite resin is close to sound teeth. Similarly, in the present study, the tooth samples restored by SFRC showed the highest fracture resistance. There was no significant difference between the SFRC group and the positive control group. Fracture resistance values in the SFRC group were found to be significantly higher than those in the GWF and indirect composite resin groups. It was found that the difference between the fracture resistance values obtained in the SFRC group and flowable bulk-fill and direct composite groups was not statistically significant. Despite the fibre content, the lowest fracture resistance was observed in the samples in the GWF group. Therefore, the null hypothesis was rejected.

In this study, the difference between the indirect and direct composite resin groups in terms of fracture strength values was not significant. Similarly, in vivo studies reported that there is no significant difference between the survival rates of direct and indirect posterior resin composite restorations [15, 26, 27]. Polymerization shrinkage is a factor that significantly affects the success of coronal restorations. To solve this problem, it is recommended to use low-viscosity composite resins in order to reduce or buffer the stress that comes with occlusal pressure [28–30]. In this study, we think that the bulk-fill composite resin, which is used as a base material under the nanohybrid composite, can be a factor in the occurrence of high fracture resistance values by absorbing occlusal pressures. Belli et al. [23] reported that the application of composite resin together with polyethylene woven fibre (PWF) increases the fracture resistance, whereas sufficient fracture resistance cannot be achieved in ETT restored with conventional posterior resin composites. Similar to this study, Kemaloglu et al. [13] claimed that both resin composite restorations reinforced with shortened fibres and woven fibres were more successful than traditional resin composites in ETT with large MOD cavities. Both Belli et al. and Kemaloglu et al. showed that the polyethylene woven fibre (PWF) placed on the cavity base and walls significantly increases the fracture resistance. However, in this study, it has been determined that the application of woven fibre does not increase the fracture resistance. While the finding that SFRC application increases fracture resistance in ETT is similar to that of Kemaloglu et al. [13], we think that the opposite result encountered in woven fibre application is due to the fibre types used. Kemaloglu et al. [13] used polyethylene fibre in their studies, while the glass fibre was used in this study. Restorations reinforced with polyethylene fibre have been reported to be more durable than those reinforced with glass fibre [31]. However, when the types of fractures in the same study are evaluated, favorable fracture percentages are higher in glass fibre-reinforced restorations than polyethylene fibre-reinforced ones. In this study, the percentage of restorable or favourable fractures in composite restorations applied without fibre usage is 10%, while it is 25% in glass fibre-reinforced restorations. This can be explained by the results of a research study conducted by Vallitu [32]. The researcher claimed that the distribution of the fibre in the resin matrix determines its physical properties. Although the three-dimensional structure of the polyethylene fibre strengthens the polymer bidirectionally, the glass woven fibre enables reinforcement in one direction due to its anisotropic feature. This creates weak areas in fibre-reinforced structures depending on the direction of the fracture forces. In addition, Huang et al. [33] investigated the physical properties of glass fibre used in composite resin reinforcement and reported that the addition of the strip-shaped fibre provides a more effective reinforcement than the woven form. In this study, while StickNET woven fibre, which gives lower values in mechanical tests, consists of glass fibres, EverX Posterior, which shows high physical properties, is composed of shortened strip-shaped fibres which are added to the composite. Yaşa et al. [27] reported that SFRC has better fracture resistance and flexure strength values, as well as shows lower polymerisation shrinkage values against bulk-fill composites. In addition, when the types of fractures were evaluated, it was claimed that the use of fibres preserved the remaining tooth structure and caused more repairable fractures than other groups. Low polymerisation shrinkage observed in SFRC may be a factor that contributes to the high fracture resistance observed in SFRCs in this study which shows the great parallelism with our study findings in terms of fracture strength, flexural strength, and restorable fracture percentages. Low polymerisation shrinkage may reduce the tension occurring in the cavity wall, thereby reducing tubercular deflection and microcracks on the walls. Therefore, durable restorations can be formed for fracture resistance tests [15, 34].

The mechanical properties of a product depend on the composition of the material. Filler content and filler properties are known to affect the mechanical properties of resin composites [35]. It is claimed that fibre-reinforced composites have sufficient flexural strength and modulus of elasticity against functional forces in the mouth [32]. Similarly, in this study, the highest flexural strength was observed in the SFRC group. While the values obtained in the SFRC group were found significantly higher than GWF, direct composite, and indirect composite groups, the difference was not significant when SFRC was compared with the flowable bulk-fill group. Although the lowest values were obtained in the GWF group, the difference between the GWF group and the direct and indirect composite groups was not significant. Ellakwa et al. [36] showed that the flexural strength of fibre-reinforced composite resins was significantly higher than that of the fibre-free group. Bae et al. [37] examined the flexural strength of composite resin samples reinforced with different fibre types and reported that the flexural strength increased significantly in all fibre-reinforced samples regardless of the fibre type added. In this study, the significant increase in the flexural strength of composites supported with shortened glass fibre is supported by the results of studies that advocate the positive effects of glass fibre reinforcement [33]. However, there was no significant increase in the flexural strength of the samples supported with GWF. The big difference observed between SFRC and GWF in terms of flexural strengths may be related to the distribution of fibres in the composite mass. In the SFRC group, glass fibres are distributed evenly within the composite mass, while fibres are concentrated only at the bottom of the composite in the GWF restoration group. In addition, no significant difference was found between the SFRC group and the bulk-fill composite resin group (*p* > 0.05). This may be due to the high flexural strength feature of the flowable resin composite material which both bulk-fill composite resin and SFRC have.

When the data of the fracture resistance on the restorations and the flexural strength of the materials are comparatively analysed, it is seen that the restorations made with SFRC, which shows the highest flexural strength, show the highest fracture strength, and the GWF showing the lowest flexural strength likewise exhibits the lowest fracture strength values. According to these results, it can be concluded that fractures are observed more frequently in restorations with materials with low flexural strength.

## 5. Conclusions

Within the limits of this in vitro study, the following results have been achieved:

- MOD cavity preparation in ETT reduces the fracture strength by 85%
- SFRC, which gives the best results in this study, can be used to increase the fracture resistance of ETT
- GWF group presented the worst direct restoration opinion in terms of fracture strength and flexural strength
- Flexural strength and MOE values of restorative materials play important roles in fracture resistance of restorations

## Figures and Tables

**Table 1:** Fracture resistance values and standard deviations.

Group 1 (DC)	Group 2 (GWF)	Group 3 (SFRC)	Group 4 (FBFC)	Group 5 (IC)	Negative control (NC)	Positive control (PC)
1938.02 (±281.62)^b,c,d^^*∗*^	1687.01 (±195.86)^d^	2228.35 (±332.5)^b^	2036.4 (±211.6)^b,c^	1903.3 (±441.3)^c,d^	382.8 (±113.2)^e^	2721.27 (±322.6)^a^

**Table 2:** Distribution of favorable and unfavorable fractures by groups.

Fracture types/groups	Group 1 (DC) (%)	Group 2 (GWF) (%)	Group 3 (SFRC) (%)	Group 4 (FBFC) (%)	Group 5 (IC) (%)
Favorable	25	10	50	45	30
Unfavorable	75	90	50	55	70

**Table 3:** Flexural strength values and standard deviations.

Group 1 (DC)	Group 2 (GWF)	Group 3 (SFRC)	Grup 4 (FBFC)	Group 5 (IC)
150.15 (±15.73)^b^	140.36 (±22.0)^b^	184.08 (±28.05)^a^	173.98 (±20.9)^a^	150.52 (±15.88)^b^

**Table 4:** Elasticity of modulus values (GPa) and standard deviations.

Group 1 (DC)	Group 2 (GWF)	Group 3 (SFRC)	Group 4 (FBFC)	Group 5 (IC)
9.73 (±0.87)^b^	8.87 (±1.7)^b^	18.89 (±3.21)^a^	6.33 (±1.37)^c^	8.3(±0.99)^b,c^

## Data Availability

The research data used to support the findings of this study are included within the article.
